# Efficacy of Low-dose Olanzapine in Combination with Sertraline on Negative Symptoms and Psychosocial Functioning in Schizophrenia: A Randomized Controlled Trial

**DOI:** 10.2174/1570159X21666230913152344

**Published:** 2023-09-19

**Authors:** Meihong Xiu, Lei Zhao, Qianqian Sun, Xiaoe Lang

**Affiliations:** 1 Peking University HuiLongGuan Clinical Medical School, Beijing HuiLongGuan Hospital, Beijing, China;; 2 Qingdao Mental Health Center, Qingdao, China;; 3 Department of Psychiatry, First Hospital of Shanxi Medical University, Taiyuan, China

**Keywords:** Schizophrenia, olanzapine, first-episode, sertraline, efficacy, negative symptoms, psychosocial functioning

## Abstract

**Background:**

Evidence for the efficacy of a low dose of olanzapine (OLA) in combination with antidepressants has been limited and without positive trials in first-episode (FE) patients with schizophrenia (SCH). This study aimed to compare the efficacy in treating negative and depressive symptoms between those FE patients with SCH treated with a combination of OLA plus sertraline and those treated with OLA monotherapy.

**Methods:**

One hundred and ninety-six first-episode and drug naïve patients with SCH were randomized to receive low-dose OLA (7.5-10 mg/day) combined with sertraline (50-100 mg/day) (OS group) or normal-dose OLA monotherapy (12.5-20 mg/day) (NO group). Clinical symptoms were evaluated by the Positive and Negative Syndrome Scale (PANSS), and the depressive symptoms were evaluated by the Hamilton Depression Scale (HAMD). Psychosocial functioning was assessed by the Personal and Social Performance Scale (PSP).

**Results:**

In the intent-to-treat efficacy analysis, the OS group had greater decreases in negative and depressive symptoms (*p*_all_ < 0.01) and a greater increase in PSP total score compared with the NO group (*p* < 0.01). Moreover, reductions in HAMD total score and PANSS negative subscore and sex were associated with the improvements in psychosocial functioning from baseline to week 24, after controlling for baseline psychosocial function, age, and onset age.

**Conclusion:**

This study demonstrates that low-dose OLA in combination with sertraline had clinically meaningful improvements not only in the negative and depressive symptoms but also in psychosocial functioning in patients with FE-SCH, while not affecting positive symptoms.

## 1 INTRODUCTION

Schizophrenia (SCH) is a chronic and debilitating disease with a lifetime prevalence of 1% in the general population [1]. Patients with SCH usually exhibit psychotic features as well as impairments in multiple domains of cognitive functions and social functioning. According to a recent Global Burden of Disease study, SCH ranked highest among 235 physical and psychiatric disorders in terms of function burden [2]. Even though current antipsychotics are commonly effective to alleviate certain psychotic symptoms related to SCH (*e.g*. hallucinations and delusions), efficacy is limited for negative symptoms and psychosocial functioning [3].

Based on data from clinical trials, real-world settings, meta-analyses, and pooled analyses, OLA is one of the most studied antipsychotics with proven efficacy in treating SCH [4-9]. It has strong inhibitory action *in vitro* at dopamine D_1_, D_2_, D_4_, serotonin (5-HT) 5-HT_2_A, 5-HT_2_C, histamine H_1_, and α_1_-adrenergic receptors, the important neurotransmitters involved in etiology and pathophysiology of SCH [10, 11]. Its structural characteristics and pharmacological effects are similar to those of the atypical antipsychotic clozapine, and its tolerance is better than that of the typical antipsychotic haloperidol. Compared to typical antipsychotics, OLA produced differential effects on the nigrostriatal and mesolimbic systems within the central nervous system and was associated with noticeably fewer adverse movement problems and a low induction of extrapyramidal symptoms [12, 13].

Although OLA treatment can significantly improve the positive symptoms of patients with SCH, there is clear evidence that it improves negative symptoms to a smaller extent than positive ones. Negative symptoms of SCH refer to the absence or diminution of normal emotional and mental activities, and the ability for independent action [14, 15], which hampers the patients’ recovery and has a negative impact on their life quality [16]. In current clinical practice, recovery for SCH patients with negative symptoms has remained elusive. Antidepressant has been a common choice for the treatment of negative symptoms in patients with SCH because of their pharmacological mechanism involving related neurotransmitters in the aetiological hypotheses of negative symptoms [17]. In addition, previous studies also support that antidepressants along with antipsychotic drugs were more efficacious in treating the negative symptoms of SCH than antipsychotic monotherapy [18-20]. Taking into account the limited efficacy of OLA on negative symptoms for patients with SCH, a combination with antidepressants is a good choice to alleviate negative symptoms of SCH.

We hypothesized that a low daily dose of OLA combined with sertraline would be superior to a standard dose of OLA in alleviating negative symptoms and improving psychosocial functioning in first-episode (FE) SCH patients. Thus, this clinical trial was designed to investigate the efficacy after 24 weeks of treatment and solve the following questions. 1) Whether there is a difference in the efficacy between the low-dose OLA combined with the sertraline group (OS) and the OLA monotherapy group (NO group)? 2) Whether there is favorable efficacy in the psychosocial functioning in the OS group compared with the NO group? and 3) Whether there is a difference in terms of improvements in depressive symptoms between the two groups?

## 2 METHODS

### 2.1 Patients

We included outpatients diagnosed with SCH by DSM-IV according to the inclusion and exclusion criteria. All recruited patients were drug-naïve FE patients with SCH. The inclusion and exclusion criteria were kept to the minimum required to be able to address the questions in this study. Inclusion criteria included: (1) SCH confirmed by the Structured Clinical Interview for DSM-IV (SCID); (2) age of 15-45 years; (3) less than five years of disease duration; (4) less than 2 weeks of lifetime antipsychotic use; and (5) ability to sign the informed consent. Exclusion criteria included: (1) major psychiatric illnesses diagnosis other than SCH; (2) current or a history of substance abuse except for nicotine and alcohol; (3) major somatic comorbidity; (4) abnormal routine biochemical measurements; and (5) a history of intolerance to OLA.

This study was conducted at First Hospital of Shanxi Medical University. The study procedures and protocol were approved by the Ethics Committee of First Hospital of Shanxi Medical University. Written informed consent was provided by the individual participant after the cluster randomization.

### 2.2 Study Design

This was a randomized open-label study conducted over 6 months. Participants were randomly in a 1:1 ratio assigned to receive low-dose OLA (7.5-10 mg/day) combined with sertraline (50-100 mg/day) in the OS group or normal-dose OLA monotherapy (12.5-20 mg/day) in the NO group.

Randomization occurred *via* a web-based system, with a unique randomization number assigned to each participant for use in case report forms. An independent investigator made random allocation cards using computer-generated random numbers. The randomization procedure was described in our previous study [20]. This clinical trial consisted of 5 visits, conducted at the end of weeks 0, 4, 8, 12, and 24. Nurses ensured treatment adherence during the study period. Clonazepam (2-3 mg/d) was permitted to treat sleep problems and relieve acute agitation during the early phase of treatment.

### 2.3 Outcomes

The primary measure outcome was clinical symptoms assessed at baseline and the end of weeks 4, 8, 12, and 24 by the Positive and Negative Syndrome Scale (PANSS) [21] and the Hamilton Depression Rating Scale (HAMD) [22] and the Clinical Global Impressions-S (CGI-S) [23]. The endpoint of outcomes was the difference in patients with SCH in the OS group *versus* those in the NO group in the pre- *versus* after 24 weeks of treatment in both groups.

The secondary outcome was the Personal and Social Performance Scale (PSP) [24], which was also assessed at baseline and 4 follow-up time points.

### 2.4 Statistical Analysis

All efficacy analyses were conducted for an intent-to-treat (ITT) analysis set that included the patients who received at least 4 weeks of treatment and had a valid baseline and at least 1 valid post-treatment follow-up.

The treatment efficacy on the primary and secondary measures between the two groups was analyzed by using a repeated-measures analysis of variance (RMANOVA). In the group comparison model of endpoint differences, clinical symptoms and psychosocial functioning assessed over 5 time points were added as the dependent variable, treatment group (OS *vs*. NO groups) was added as the independent variable, and sex, age, and onset age were covariates. We focused more on the interaction effect of time by treatment group. If there was a significant interaction effect, we then ran an analysis of covariance (ANCOVA) with the measurements at baseline as covariates to examine the differences in the different efficacy endpoint measures (Week 4, 8, 12, and 24) between the OS and NO groups. Additionally, ANCOVA was also conducted to compare the changes of primary and secondary measures from baseline to the different endpoints between the two groups.

Linear regression analysis was used to investigate the potential key factors associated with the improvement in psychosocial functioning. The following variables were included in this regression model: baseline psychosocial functioning, sex, age, age at onset, and the reductions in clinical symptoms.

Data were analyzed using SPSS software Version 22.0. Multiple tests correction was performed by the Bonferroni method. Significance was set at *p* < 0.05 for all analyses.

## 3 RESULTS

### 3.1 Patient Characteristics

Of the 206 SCH patients screened, 196 patients met the inclusion and exclusion criteria. All patients were randomized to the OS group (n=98) and the NO group (n=98). A total of 11 patients withdrew their consent on the first visit. Eighteen patients dropped out in the follow-up (n=6 in the OS group and n=12 in the NO group). In the NO group, 9 patients discontinued for side effects caused by antipsychotics (*e.g*. weight gain, abnormal blood sugar, and lipids), and 3 patients were lost to follow-up. In the OS group, 2 patients discontinued for side effects caused by antipsychotics (*e.g*. weight gain and abnormal blood lipids), and 1 patient was lost to follow-up. The rate of dropout was higher in the NO group (n=12) than in the OS group (n=6) (Fig. **S1**). A total of 185 were included in the efficacy ITT-population analysis.

At baseline, there were no significant differences in age, sex, duration of illness, and onset age between the OS and NO groups (P_all_>0.05). Yet, we found that individuals in the OS group had higher scores in negative symptoms, general psychopathology, the PANSS total score, and HAMD than the NO group (P_all_<0.05) (Table **1**).

### 3.2 Efficacy

**Fig. (1):**
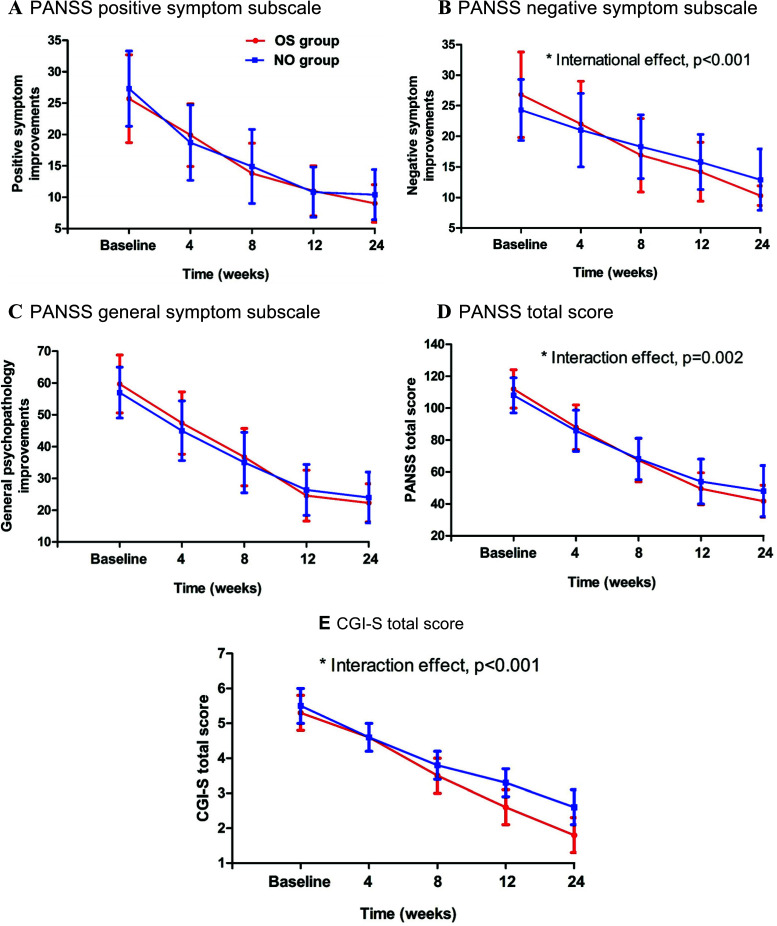
Mean Changes in the Positive and Negative Syndrome Scale (PANSS) total Score and Clinical Global Impression-Severity of Illness (CGI-S) and mean changes in the positive and negative symptom and general subscales. **Note**: *p < 0.01 compared with control group by a repeated-measures analysis of variance (RMANOVA).

**Fig. (2):**
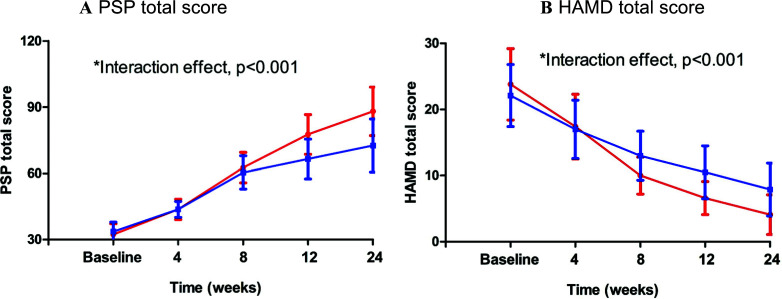
Mean changes in the Psychosocial Functioning (PSP) and the Hamilton Depression Rating Scale (HAMD). **Note**: *p < 0.001 compared with control group by a repeated-measures analysis of variance (RMANOVA).

Treatment with OLA combined with sertraline demonstrated statistically significant changes in PANSS negative symptoms (Fig. **1B**), PANSS total score (Fig. **1D**), and CGI-S scores (Fig. **1E**) after Bonferroni correction (p_Bonferroni_<0.01). In addition, HAMD was also significantly decreased after treatment in both groups, and the OS group showed a significant difference from the NO group (p_Bonferroni_ <0.01) (effect size, from 0.63 to 4.02) (Table **2**) (Fig. **2**). In RMANOVA analysis, significant interaction effects of the group by the time were observed in PANSS total score, negative symptom subscore, CGI-S scores, and HAMD total score (p_all_<0.001). Statistically significant differences from the control group in these clinical symptoms were observed at the week 4 assessment and continued through the week 24 assessment with combination therapy (Fig. **1B**, **D**, and **E**). Additionally, there were significant differences in the clinical symptoms from week 4 and continued through week 24 in the OS group. However, we did not find any significant interaction effect on positive symptoms or general psychopathology (Figs. **1A** and **1C**).

Furthermore, patients in the OS group also met another key secondary endpoint with a significant change in PSP score from baseline to week 24 *versus* the control group (mean difference: 14.5, 95% CI: 13.7 to 16.0).

In addition, we found that patients in the OS group had fewer adverse effects as measured by the Treatment Emergent Symptom Scale (TESS) (Table **3**).

### 3.3 Related Factors with Social Function Improvement

We further analyzed the factors related to the improvements in psychosocial functioning. Correlation analysis found that there were significant positive associations between the increase in PSP total score and the reductions in PANSS negative symptom from baseline, PANSS total score, CGI total score, and HAMD total score with an r-value ranging from 0.24 to 0.45 (p_all_<0.01). Linear regression analysis confirmed the association between the improvement of psychosocial function and the reductions in PANSS negative symptoms (β = 4.3, t = 7.8, *p* < 0.001), HAMD total score (β = 5.4, t = 7.8, *p* < 0.001), CGI total score (β = 5.4, t = 7.8, *p* < 0.001), after controlling for sex, age, onset age, and baseline PSP total score (*R*^2^ = 0.60).

## 4 DISCUSSION

This study found that compared with standard-dose OLA monotherapy, low doses of OLA plus sertraline significantly reduced negative symptoms and increased psychosocial functioning commonly observed with antipsychotic treatment in patients with FE-SCH. In addition, the efficacy of low doses of OLA in combination with sertraline on positive symptoms of SCH was comparable to that of standard dose OLA monotherapy.

Treatment with low-dose OLA in combination with sertraline significantly improved negative symptoms, as assessed with the PANSS scale relative to OLA monotherapy in patients. This finding is in line with previous studies as well as the meta-analyses, suggesting that sertraline as an add-on combination treatment to antipsychotics was more effective in treating negative symptoms than antipsychotic monotherapy [18]. The statistically significant overall effect size of 0.92 for the combination therapy with sertraline may be considered large using Cohen’s guidelines for the magnitude of the effect size [25]. Interestingly, in line with other studies, sertraline add-on to OLA was effective in improving depressive symptoms in SCH patients [20, 26-29]. A previous study using combination pharmacotherapy with a standard dosage of OLA (15-20 mg/day) and sertraline to treat patients with depressive symptoms found that combination therapy was associated with higher remission rates and was comparably superior in clinical symptoms than OLA monotherapy [30]. A possible explanation for the reported clinical efficacy is associated with the modulatory effect on the 5-HT system of sertraline for negative symptoms and depressive symptoms, as dysfunctions of dopaminergic and 5-HT neurons have been linked to SCH. Additionally, sertraline appears to improve the negative symptoms by inhibiting dopamine transporters and reuptake and by enhancing central dopaminergic neuron activity [31, 32]. Considering not only the statistical significance but also its clinical significance, a low dose of OLA combined with sertraline for negative and depressive symptoms in patients with SCH may appear justified.

It should be noted that this study found that combination therapy of low-dose OLA showed comparable efficacy for positive symptoms of SCH compared with standard dose monotherapy. Although previous studies reported a higher dose of OLA provided more efficacious benefits for patients compared to the usual dose range recommended in the protocol [33], our findings demonstrate that decreased dose of OLA showed no impact on the efficacy of positive symptoms relative to a usual dose of OLA. We speculate that it may be due to the modulatory effect of sertraline on transmitters in the combination therapy, and then the effect on the negative and depressive symptoms [34]. This study provides a new combination of the antipsychotic regimen used to treat several domains of clinical symptoms of SCH.

More importantly, this study reported a better efficacy of the combination pharmacotherapy for psychosocial functioning. The positive findings must be considered regarding the reductions in negative and depressive symptoms by sertraline, which has been confirmed by the regression analysis. The beneficial effects of sertraline on depressive symptoms may be the potential mechanism of the advantages of this promising combination treatment strategy for patients with SCZ. Patients with SCH often show deficits in several domains of psychosocial functioning [35-38], and as a result, they are usually at high risk of unemployment, less likely to engage in social activities, and have no or few satisfying intimate relationships [39]. Our study demonstrates that add-on antidepressants are an important aspect in the effective clinical management of depressive and negative symptoms, because additional symptom relief may help to increase psychosocial functioning and improve quality of life in patients with FE-SCH.

Some limitations should be noted in this study. First, this was a short-term cohort study. A 24-week treatment duration may not address the maintenance of efficacy, which should be assessed after long-term combination therapy. Second, it was not a placebo-controlled, double-blinded clinical trial. Although the nurses did not tell the patients which medications they were taking, the patients may have identified the intervention as the OS group took two capsules of medication and the control group took one. Third, SCH patients recruited in this study had to meet specific inclusion and exclusion criteria, which may limit the generalization of the findings to a broader population.

## CONCLUSION

Despite the limitations, this is the first trial to suggest that FE-SCH patients on low doses of OLA in combination with sertraline showed greater improvements in negative symptoms and psychosocial functioning than usual doses of OLA monotherapy. Furthermore, the efficacy of combination treatment was comparable in terms of positive symptoms relative to OLA medication alone. Previous combination therapy with the usual dosage incurred more adverse effects and higher costs. Our study of low-dose OLA combined with antidepressants provides a promising treatment option for the early stages of SCH, which may benefit patients in clinical practice.

## Figures and Tables

**Table 1:** Demographic and clinical characteristics data in combination therapy group (OS) and control group (NO) (mean ± SD).

**Variable**	**OS Group (n = 98)**	**NO Group (n = 87)**	**F or *X*^2^ (*p* Value)**
Gender (male/female)	52/46	40/47	0.9(0.34)
Age (years)	24.5 ± 7.1	24.0 ± 6.4	0.2(0.62)
Age of onset (years)	21.9 ± 6.0	21.9 ± 5.8	0.002(0.96)
Duration of illness (years)	2.5 ± 1.7	2.0 ± 1.5	3.1(0.08)
P	25.8 ± 6.9	27.2 ± 6.7	2.1(0.15)
N	26.8 ± 7.4	24.4 ± 5.8	**5.7(0.02)**
G	59.6 ± 8.9	56.8 ± 8.4	**4.8(0.03)**
PANSS total score	112.1 ± 12.4	108.4 ± 11.5	**4.4(0.04)**
HAMD total score	23.6 ± 5.5	22.1 ± 5.0	**4.0(0.048)**
PSP total score	32.3 ± 4.9	33.6 ± 4.3	2.9(0.09)
CGI-S total score	5.3 ± 0.5	5.5 ± 0.5	3.7(0.06)

**Note:** *SD* Standard Deviation, *P* positive subscore, *N* negative subscore, *G* general psychopathology subscore, *CGI-S* Clinical Global Impression-severity, *PSP* Personal, and Social Performance Scale, *HAMD* Hamilton Depression Rating Scale.

**Table 2:** PANSS scores, HAMD score, CGI-S and PSP at baseline, week 4, week 8, week 12 and week 24 in patients treated with OLA plus sertraline (OS) group and OLA monotherapy (NO) group (mean ± standard deviations).

**-**	**Baseline**	**Week 4**	**Week 8**	**Week 12**	**Week 24**	**Group Effect F (*p* Value)^a^**	**Interaction Effect F(*p* Value)^a^**
**PANSS Positive Subscore**						1.1(0.29)	6.1(0.014)
OS group	25.8 ± 6.9	19.9 ± 5.8	14.1 ± 5.0	10.8 ± 3.9	9.2 ± 3.2	-	-
NO group	27.2 ± 6.7	18.7 ± 6.3	15.5 ± 6.1	11.7 ± 4.7	10.4 ± 4.5	-	-
**PANSS Negative Subscore**						2.2(0.14)	27.3(<0.001)
OS group	26.8 ± 7.4	22.0 ± 7.2	17.1 ± 6.2	14.2 ± 5.0	10.3 ± 3.3	-	-
NO group	24.4 ± 5.8	22.0 ± 6.0	18.7 ± 5.6	15.8 ± 5.4	12.9 ± 5.1	-	-
**PANSS General Psychological Subscore**						0.002(0.98)	5.7(0.02)
OS group	59.6 ± 8.9	47.1 ± 9.8	37.0 ± 9.3	24.6 ± 8.0	22.3 ± 5.8	-	-
NO group	56.8 ± 8.4	44.7 ± 9.6	36.1 ± 10.1	26.4 ± 8.0	24.0 ± 8.7	-	-
**PANSS Total Score**						2.6(0.11)	10.1(0.002)
OS group	112.1 ± 12.4	88.1 ± 14.4	68.3 ± 14.0	49.5 ± 10.6	41.7 ± 11.2	-	-
NO group	108.4 ± 11.5	85.5 ± 13.2	70.3 ± 14.5	53.9 ± 14.1	47.2 ± 16.1	-	-
**CGI-S Score**						38.1(<0.001)	76.1(<0.001)
OS group	5.3 ± 0.5	4.6 ± 0.5	3.5 ± 0.5	2.6 ± 0.6	1.8 ± 0.6	-	-
NO group	5.5 ± 0.5	4.6 ± 0.5	3.7 ± 0.4	3.3 ± 0.5	2.6 ± 0.5	-	-
**HAMD Total Score**						24.6(<0.001)	32.1(<0.001)
OS group	23.6 ± 5.5	17.2 ± 5.0	10.3 ± 3.0	6.6 ± 2.7	4.1 ± 3.1	-	-
NO group	22.1 ± 5.0	16.9 ± 4.5	13.0 ± 4.0	10.5 ± 4.0	7.9 ± 4.2	-	-
**PSP Total Score**						50.5(<0.001)	69.7(<0.001)
OS group	32.5 ± 5.0	43.8 ± 4.5	62.6 ± 6.9	77.6 ± 9.4	88.1 ± 11.5	-	-
NO group	33.9 ± 4.4	43.8 ± 4.5	60.4 ± 7.5	66.5 ± 9.4	72.6 ± 12.0	-	-

**Note:** ^a^Adjusted *F* value controlling for sex, age and onset age.

**Table 3:** Comparison of adverse effects using treatment emergent symptom scale between the OS group and NO group.

**-**	**OS Group ** **(n = 98)**	**NO Group ** **(n = 87)**
Adverse events	n (%)	n (%)
Tremor	3(3.1)	3(3.5)
Akathisia	2(2.0)	7(8.2)
Insomnia	2(2.0)	1(1.2)
Somnolence	4(4.1)*	18(21.2)
Constipation	5(5.1)	8(9.4)
Nausea	4(4.1)	3(3.5)
Dizziness	5(5.1)	7(8.2)
Weight gain	28(28.6)*	85(97.7)
Electrocardiographic changes	1(1.0)	3(3.5)
Muscle rigidity	1(1.0)	2(2.4)
Dry mouth	3(3.1)	3(3.5)

**Note:** *p < 0.05.

## Data Availability

The datasets generated and analyzed during the current study are available from the corresponding author upon reasonable request.

## LIST OF ABBREVIATIONS

CGI-S: Clinical Global Impressions-S
HAMD: Hamilton Depression Rating Scale
ITT: Intent-to-Treat
PANSS: Positive and Negative Syndrome Scale
PSP: Social Performance Scale
SCH: Schizophrenia
TESS: Treatment Emergent Symptom Scale
