# Supplementary material for: Efficacy of Low-dose Olanzapine in Combination with Sertraline on Negative Symptoms and Psychosocial Functioning in Schizophrenia: A Randomized Controlled Trial
Source: Curr Neuropharmacol. 2023 Sep 19;22(8):1406–13. doi: 10.2174/1570159X21666230913152344 (PMC11092916; doi:10.2174/1570159X21666230913152344)
Supplement: Supplementary file 1 [file CN-22-1406_SD1.pdf]

Supplementary Material

Efficacy of Low-dose Olanzapine in Combination with Sertraline on Negative Symptoms and Psychosocial Functioning in Schizophrenia: A Randomized Controlled Trial

Meihong Xiu[1], Lei Zhao[2], Qianqian Sun[2] and Xiaoe Lang[3,*]

[1]*Peking University HuiLongGuan Clinical Medical School, Beijing HuiLongGuan Hospital, Beijing, China;* [2]*Qingdao Mental Health Center, Qingdao, China;* [3]*Department of Psychiatry, First Hospital of Shanxi Medical University, Taiyuan, China*

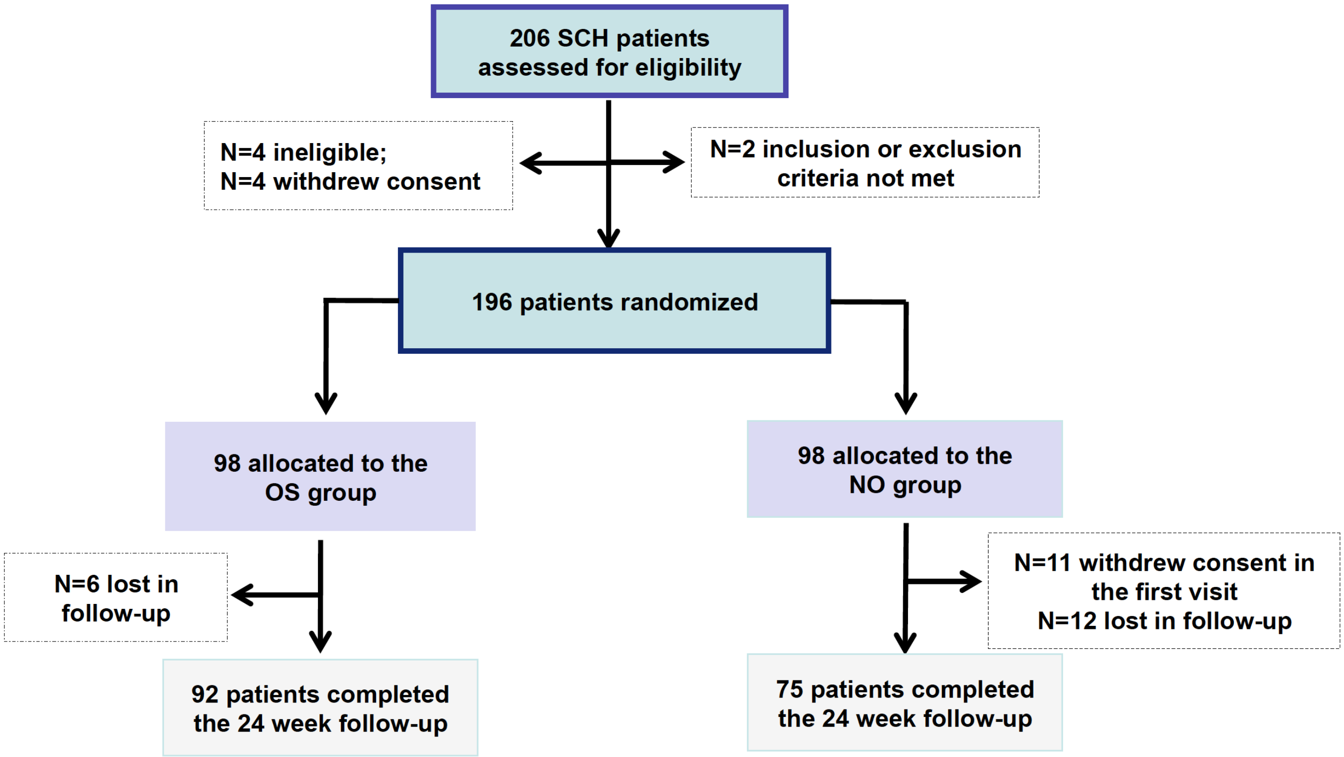

Fig. (S1).

1875-6190/24 $65.00+.00 © 2024 Bentham Science Publishers
